# Lifestyle advice for hypertension or diabetes: trend analysis from 2002 to 2017 in England

**DOI:** 10.3399/BJGP.2021.0493

**Published:** 2022-03-08

**Authors:** John A Henry, Susan A Jebb, Paul Aveyard, Cesar Garriga, Julia Hippisley-Cox, Carmen Piernas

**Affiliations:** Nuffield Department of Primary Care Health Sciences, University of Oxford, Oxford.; Nuffield Department of Primary Care Health Sciences, University of Oxford, Oxford.; Nuffield Department of Primary Care Health Sciences, University of Oxford, Oxford.; Nuffield Department of Primary Care Health Sciences, University of Oxford, Oxford.; Nuffield Department of Primary Care Health Sciences, University of Oxford, Oxford.; Nuffield Department of Primary Care Health Sciences, University of Oxford, Oxford.

**Keywords:** diabetes mellitus, hypertension, lifestyle advice, primary health care, weight loss

## Abstract

**Background:**

Guidelines recommend that GPs give patients lifestyle advice to manage hypertension and diabetes. Increasing evidence shows that this is an effective and practical treatment for these conditions, but it is unclear whether GPs offer this support.

**Aim:**

To investigate trends in the percentage of patients with hypertension/diabetes receiving lifestyle advice versus medication.

**Design and setting:**

This was a trend analysis of self-reported data from the annual Health Survey for England (HSE) (2003–2017) and GP-recorded data from the QResearch database (2002–2016).

**Method:**

The percentage of patients with hypertension or diabetes who received lifestyle advice or medication was calculated in each year. Associations between likelihood of receiving lifestyle advice and characteristics were assessed using multivariable logistic regression.

**Results:**

The percentage of patients receiving lifestyle advice was consistently lower than those receiving medication in both self-reported and medical records. There was consistent evidence of increasing trends in the percentage of patients with hypertension receiving lifestyle advice (HSE 13.8% to 20.1%; *P_trend_*<0.001; QResearch 11.0% to 22.7%; *P_trend_*<0.001). For diabetes, there was a non-significant decline in self-reported receipt of lifestyle advice (45.0% to 27.9%; *P_trend_* = 0.111) and a significant increase in medically recorded delivery of this advice (20.7% to 40.5%; *P_trend_*<0.001). Patients with hypertension who were overweight or obese were more likely to receive lifestyle advice than those of a healthy weight, whereas the opposite was true for diabetes.

**Conclusion:**

Only a minority of patients with diabetes or hypertension report receiving lifestyle advice or have this recorded in their medical records. Interventions beyond guidelines are needed to increase the delivery of behavioural interventions to treat these conditions.

## INTRODUCTION

Hypertension and diabetes are prevalent and costly conditions,^1^ and behavioural risk factors contribute significantly to the development of both.^2^^,^^3^ There is good evidence that weight loss, increased physical activity, alcohol reduction, and smoking cessation can help treat both hypertension and diabetes.^4^^–^7 For example, with behavioural support programmes shown to be effective when delivered in a routine care context it is possible to achieve diabetes remission or have effects large enough to de-escalate medication for hypertension.^8^^–^10 Moreover, clinical trials show that, when clinicians endorse, offer, and facilitate referrals to these programmes, this is highly acceptable to patients and leads to high uptake.^6^

Research from several countries (UK, US, the Netherlands, and Ghana) has generally shown that a minority of patients with hypertension or diabetes receive lifestyle advice to help manage their condition.^11^^–^15 Evidence from the UK is limited, with one study showing that a third of patients received lifestyle advice for uncomplicated mild hypertension.^11^ Given the increasing focus and evidence available to support treating hypertension and diabetes through behavioural interventions, the current study aimed to examine trends in the frequency of this in the NHS, using both patient-reported data and GP-recorded data, which each offer a different perspective. For example, it is possible that patients may forget advice that was given in the past, or that the advice could be so inapparent that it is not perceived as such by patients, or advice could be given by health professionals without it being recorded. One study on smoking advice found that, before the introduction of a pay for performance programme to incentivise advice giving, the prevalence of advice recalled by patient reports closely matched that from medical records. This diverged after the introduction of the incentive, with advice recorded in medical records being more common than patients recalled.^16^ Studying data from both sources can add validity to estimates of trends and factors that predispose to the delivery of lifestyle advice. This is what the authors aimed to investigate here.

## METHOD

### Study design and population

Data from the Health Survey for England (HSE) were used; this is an annual cross-sectional survey in which information on health and health-related behaviours is collected from participants in a nationally representative sample of the English population. Further information on the survey methodology can be obtained online.^17^ The current analysis included the years 2003–2017, inclusive. Information collected varies by year and the years 2002, 2007, 2008, 2016, and 2018 were excluded as they did not contain sufficient information on lifestyle advice. General practice records from the QResearch database of anonymised health records of patients from 1500 general practices throughout England who use the EMIS clinical computer system were also used. The data held include current patients and patients who have died or left the practice. Data were extracted from the QResearch database for patients aged 40–74 years between 2009 and 2017 (that is, those who would be eligible for the English NHS Health Check Programme during this period if they did not have hypertension/diabetes)^18^ who had a diagnosis of hypertension or type 2 diabetes (Supplementary Figure S1).

**Table:** How this fits in

Guidelines recommend that clinicians offer lifestyle advice, such as weight loss, physical activity, smoking cessation, and alcohol reduction advice, to patients with hypertension or diabetes. However, it is unclear what proportion of patients receive this lifestyle advice in a primary care setting. This study shows that the proportion of patients with hypertension or diabetes receiving lifestyle advice has increased over time but remains consistently lower than those receiving medication, for hypertension and diabetes, in both patient-reported and medically recorded data. Interventions beyond guidelines are needed to improve the implementation of lifestyle modification as a treatment approach for these conditions.

### Ascertainment of hypertension and diabetes

In the HSE analysis, hypertension and diabetes were defined as self-reported ‘doctor diagnosed’ hypertension/diabetes and/or ‘currently receiving treatments/advice’, excluding hypertension/diabetes during pregnancy. The HSE makes no distinction between type 1 and type 2 diabetes, and so the term ‘diabetes’ is used when discussing the HSE data henceforth (Supplementary Table S1).

In QResearch, data from patients who had a coded diagnosis of hypertension or type 2 diabetes diagnosed between 2002 and 2016 were analysed. In this article, the term ‘type 2 diabetes’ is used when discussing QResearch data henceforth.

### Lifestyle advice and other treatments

In the HSE, individuals reported whether they were currently receiving medication for hypertension/diabetes. They were asked whether they were currently receiving any other treatments or advice for their hypertension/diabetes, and any person who reported receiving advice addressing behavioural risk factors for either hypertension or diabetes was coded as receiving ‘lifestyle advice’ (Supplementary Table S1).

In QResearch, the proportion of those with a diagnosis of hypertension/type 2 diabetes who received medication within 1 year of diagnosis was analysed (Supplementary Table S2). A composite lifestyle advice variable was again generated (Supplementary Table S3) and the proportion of those coded as receiving lifestyle advice for hypertension/type 2 diabetes in the year following diagnosis calculated.

### Sociodemographic and anthropometric characteristics

In HSE, sociodemographic variables included self-reported age, sex, social class, and ethnicity. Age was grouped into <35, 36–59, and ≥60 years. Body mass index (BMI) was calculated by trained nurses and categorised as healthy weight (BMI ≥18.5 to <25), overweight (BMI ≥25 to <30), and obesity (BMI ≥30).^19^

In QResearch, information was available on age, sex, area deprivation index (based on the Townsend score,^20^ where quintile 1 indicates the least deprived and quintile 5 indicates the most deprived), ethnicity, and BMI *.* Data on BMI for those patients who had a BMI record within 1 year of diagnosis of hypertension/type 2 diabetes were extracted. For those with multiple BMI records, the reading closest to the date of diagnosis was used.

### Statistical analyses

The proportion of adult participants (≥18 years) who reported receiving lifestyle advice or medication in each survey year was calculated; and *P*-trends were obtained by including the year variable as continuous in the model. A multivariable logistic regression model was used to investigate the likelihood of receiving ‘lifestyle advice’ in relation to participant characteristics in each dataset. In analysing HSE data, survey sampling weights and appropriate commands were used to obtain nationally representative results, corrected for non-response bias, and to account for clustering in the sampling.

In HSE, the prevalence of hypertension and diabetes was calculated each year. In QResearch, the proportion of patients who were provided with lifestyle advice and medication within 1 year following diagnosis in each study year was calculated. Stata 14SE (Stata Corp, College Station, TX) was used for all analyses. A *P*-value of less than 0.05 was deemed statistically significant.

## RESULTS

### Study population

Demographic and anthropometric characteristics of the HSE responders can be found in Supplementary Table S4 *.* From 2003 to 2017 the prevalence of hypertension increased from 23.6% to 26.1% of adults in the survey and the prevalence of diabetes increased from 4.1% to 7.2%. The prevalence of hypertension and diabetes appeared abnormally low in 2004, so this year was excluded from subsequent analyses (Supplementary Table S5). From the QResearch database, 1 507 378 patients had hypertension and 569 036 patients had type 2 diabetes and were eligible for analysis (Supplementary Figure S1).

### Hypertension

**Figure 1.**
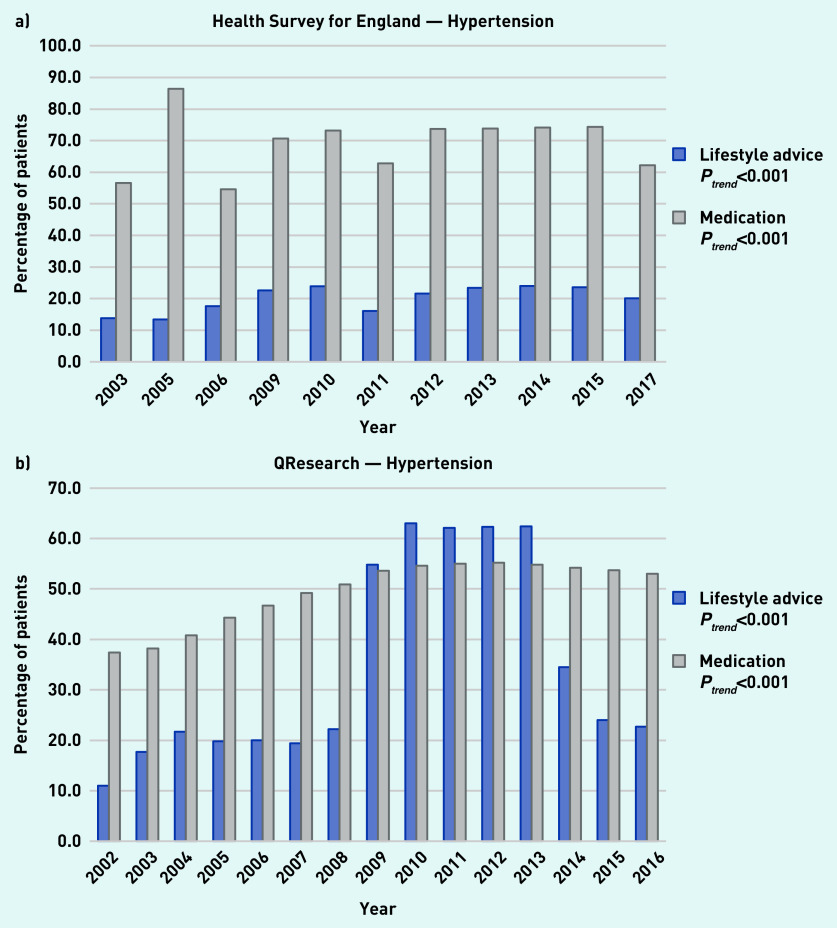
*a) Trends in the proportion of patients receiving lifestyle advice or medication for hypertension as reported in available years from the Health Survey for England from 2003 to 2017. b) Trends in the proportion of patients receiving lifestyle advice or medication for hypertension within 1 year of diagnosis as reported in the QResearch database from 2002 to 2016.*

In the HSE, the proportion of adults with hypertension who reported receiving lifestyle advice increased significantly from 13.8% in 2003 to 20.1% in 2017 and the proportion of patients reporting receiving antihypertensive medication increased significantly from 56.6% to 62.2% over the same period (Figure 1a). Analysis of medical records from QResearch showed that the proportion of adults with hypertension who received lifestyle advice increased significantly from 11.0% in 2002 to 22.7% in 2016 and the proportion receiving medication significantly increased from 37.4% to 53.0% over the same time period (Figure 1b).

A large increase in the recording of lifestyle advice was apparent between 2009 and 2013, corresponding with changes in the Quality and Outcomes Framework (QOF) around this time. Alcohol intervention support or referral was the most commonly delivered individual component of the lifestyle variable with 12.6% of patients with hypertension receiving this in 2016 (Supplementary Table S6).

### Diabetes

**Figure 2.**
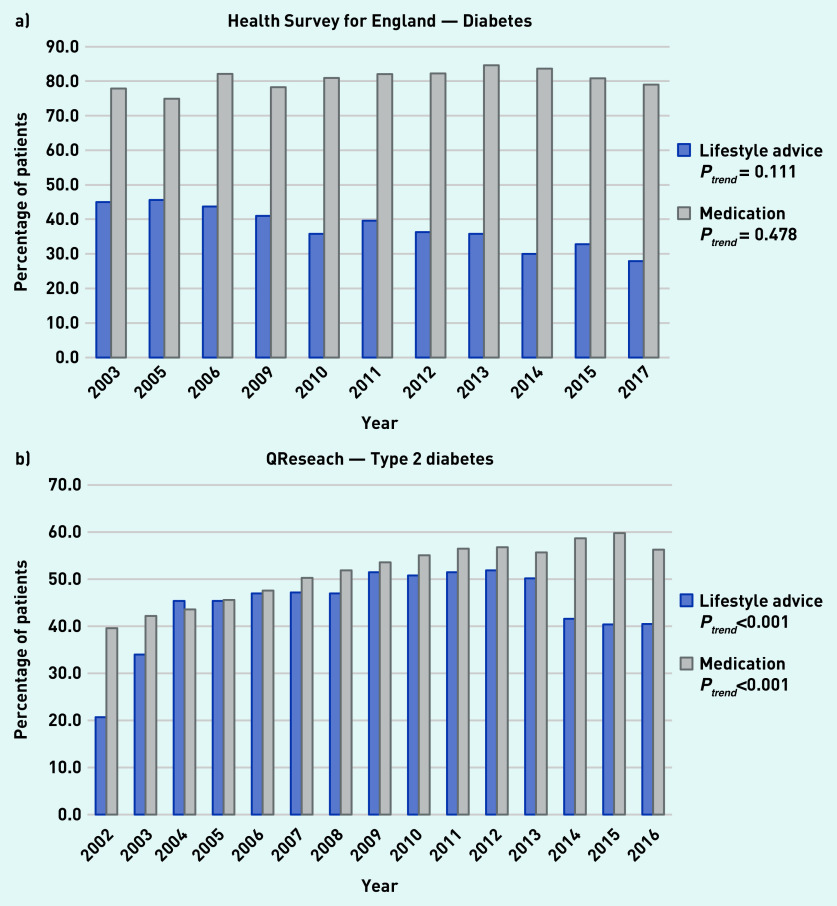
*a) Trends in the proportion of patients receiving lifestyle advice or medication for diabetes as reported in available years from the Heath Survey for England from 2003 to 2017. b) Trends in the proportion of patients receiving lifestyle advice or medication for type 2 diabetes within 1 year of diagnosis as reported in the QResearch database from 2002 to 2016.*

In the HSE, the proportion of adults with diabetes who reported receiving lifestyle advice decreased over time, although this trend was not statistically significant (45.0% to 27.9%) and the proportion of those taking medication did not change significantly (77.9% to 79.0%) (Figure 2a). Analysis of medical records from QResearch showed that the proportion of those with type 2 diabetes who received lifestyle advice increased significantly from 20.7% in 2002 to 40.5% in 2016 and the proportion receiving medication increased significantly from 39.6% to 56.3% over the same period (Figure 2b).

Weight management support or referral was the most commonly delivered type of lifestyle advice, with 23.9% of patients with type 2 diabetes receiving this in 2016 (Supplementary Table S6).

### Predictors of receiving lifestyle advice

For patients with hypertension in the HSE, adults aged under 35 were more likely to report receiving lifestyle advice than patients over 60 whereas females and patients of white ethnicity were less likely to report receiving advice than males or those from other ethnic backgrounds. Patients receiving antihypertensive medication or who were overweight were more likely to receive lifestyle advice than the comparator group (Table 1).

**Table 1.** Likelihood of receiving lifestyle advice by participant characteristics and receipt of medication, across all survey years, among adults with hypertension or diabetes included in the Health Survey for England from 2003 to 2017^a^

	**Hypertension**		**Diabetes**	

	**OR (95% CI)**	***P*-value**	**OR (95% CI)**	***P*-value**
Age, years				
36–59	0.76 (0.53 to 1.09)	0.132	0.94 (0.45 to 1.96)	0.877
≥60	0.39 (0.27 to 0.55)	<0.001	0.95 (0.46 to 1.93)	0.879

Sex				
Female	0.81 (0.70 to 0.94)	0.005	1.26 (1.02 to 1.55)	0.032

Social class				
IIIN — skilled non-manual and IIIM — skilled manual	1.01 (0.82 to 1.24)	0.953	0.86 (0.67 to 1.10)	0.241
I — professional and II — managerial technical	1.05 (0.87 to 1.26)	0.610	1.11 (0.81 to 1.53)	0.517

Ethnicity				
Black and minority ethnic	1.52 (1.02 to 2.24)	0.037	0.70 (0.47 to 1.05)	0.083

BMI				
≥25 to <30	1.61 (1.25 to 2.07)	<0.001	0.77 (0.53 to 1.11)	0.160
BMI ≥30	2.27 (1.84 to 2.80)	<0.001	0.72 (0.52 to 1.00)	0.051

a *Estimates from multivariable logistic regression model. Each coefficient shows the OR of receiving lifestyle advice with the reference category while adjusting for all other variables listed. Reference groups were aged 18–35 years, male, IV/V — manual non-skilled social class, white ethnicity, BMI 18.5 to 25 and not receiving medication, adjusted for survey year. BMI = body mass index. CI = confidence interval. OR = odds ratio.*

Similar findings were observed in QResearch, where females and patients of white ethnicity were less likely to have a record of lifestyle advice for their hypertension than males or those from other ethnic backgrounds *.* Furthermore, patients over 35 years of age, those who were receiving medication, or who were overweight were also more likely to have a record of lifestyle advice than the comparator group (Table 2). For patients with diabetes, lifestyle advice was more commonly reported by females and those not taking medication. There was a trend towards patients of white ethnicity being more likely to receive lifestyle advice and those with obesity being less likely to receive lifestyle advice, although this did not reach statistical significance in either case (Table 1).

**Table 2.** Likelihood of providing lifestyle advice by patient characteristics and receipt of medication within 1 year of diagnosis, across all survey years, among adults with hypertension or type 2 diabetes in the QResearch database from 2002 to 2016^a^

	**Hypertension**		**Diabetes**	

	**OR (95% CI)**	***P*-value**	**OR (95% CI)**	***P*-value**
Age, years				
36–59	1.17 (1.11 to 1.23)	<0.001	0.88 (0.82 to 0.95)	0.001
≥60	1.10 (1.05 to 1.16)	<0.001	0.73 (0.68 to 0.79)	<0.001

Sex				
Female	0.97 (0.96 to 0.98)	<0.001	0.99 (0.97 to 1.00)	0.058

Townsend quintile				
2	0.99 (0.97 to 1.01)	0.505	1.00 (0.97 to 1.02)	0.800
3	1.00 (0.98 to 1.02)	0.775	0.97 (0.95 to 0.99)	0.019
4	1.02 (1.00 to 1.04)	0.060	0.94 (0.92 to 0.97)	<0.001
5	1.11 (1.07 to 1.11)	<0.001	0.83 (0.81 to 0.85)	<0.001

Ethnicity				
Black and minority ethnic	1.06 (1.04 to 1.08)	<0.001	0.93 (0.91 to 0.95)	<0.001

BMI				
≥25 to <30	1.04 (1.02 to 1.06)	<0.001	0.96 (0.94 to 0.99)	0.005
≥30	1.02 (1.00 to 1.03)	0.068	0.88 (0.85 to 0.90)	<0.001

Medication				
Yes	1.47 (1.45 to 1.49)	<0.001	1.29 (1.27 to 1.31)	<0.001

a *Estimates from multivariable logistic regression model. Each coefficient shows the OR of receiving lifestyle advice with the reference category while adjusting for all other variables listed. Reference groups were aged 18–35 years, male, Townsend quintile 1, white ethnicity, BMI 18.5 to 25 and not receiving medication within 1 year of diagnosis, adjusted for year. BMI = body mass index. CI = confidence interval. OR = odds ratio.*

In the QResearch database the finding that patients of white ethnicity were more likely to receive lifestyle advice and those with obesity being less likely to receive lifestyle advice was also observed and reached statistical significance. There were also differences, however, with patients taking medication being more likely to have a record of delivery of lifestyle advice than those not taking medication *.* Recorded delivery of lifestyle advice was less common with increasing age and with increasing deprivation (Table 2).

## DISCUSSION

### Summary

Self-reported data and GP records showed that most patients with hypertension and diabetes do not receive lifestyle advice, whereas management by medication is more common. The proportion of patients receiving lifestyle advice for hypertension increased over time in both sources; however, the GP records (QResearch) appear to be dominated by a spike from 2009 to 2013 corresponding with the introduction of a QOF encouraging provision of lifestyle advice, which is not reflected in the self-reported data.

For patients with diabetes, the evidence was inconsistent, with a decline in patients reporting receiving advice but an increase in reports of advice being provided based on medical records. Associations between lifestyle advice and participant characteristics were mixed but provide evidence of differences by age, sex, ethnicity, and minor differences by socioeconomic status. For patients with hypertension who were overweight or with obesity, advice was more common, whereas for diabetes it was less common than for patients with a healthy weight.

### Strengths and limitations

To the authors’ knowledge, this is the first study using self-reported and healthcare records to examine changes in the provision of lifestyle advice for hypertension and diabetes in England. This allowed the examination of the relationship between what a healthcare professional reports delivering and what a patient reports having received. The disparities between the two sources may suggest a need for further support to aid the implementation of lifestyle advice. A strength of this analysis is large sample sizes provided by the HSE and QResearch databases with adequate power to investigate changes in trends and associations with various sociodemographic factors.

There are, however, several limitations. The fact that the results are not wholly consistent may reflect limitations in the methods employed. It is possible that not all interventions relating to lifestyle advice have been captured in the electronic healthcare records, potentially leading to an under-recording of lifestyle advice provision, for example, the current study did not include codes for referral to diabetes structured education that may include some aspects of lifestyle advice. It may also be that patients are receiving lifestyle advice from other sources such as support groups, which may not have been recorded in their medical records.^21^ Relevant information was not available for every year in the HSE (2002, 2007, 2008, 2016, and 2018 were excluded) and 60.8% and 33.0% of patients with hypertension and type 2 diabetes, respectively, in the QResearch database do not have a recent BMI recorded.

In the HSE data, the current study defined hypertension and diabetes based on self-reported data from participants, rather than direct measurements of blood pressure and glycated haemoglobin (HbA1c) obtained during a nurse visit. The QResearch population is broadly representative of the national population and thus findings ought to be similar to the data obtained from the HSE. However, the questions asked of the two populations were slightly different, with HSE participants being asked if they are currently receiving lifestyle advice whereas in QResearch this advice is recorded within 1 year of diagnosis. The ambiguity of the question asked in the HSE may lead to some under-reporting of receiving lifestyle advice, dependent on the participants’ interpretation of ‘currently receiving’. Furthermore, as the HSE does not make a distinction between type 1 and type 2 diabetes, it is likely that the populations in the diabetes analysis are subtly different. Type 1 diabetes is estimated to account for around 8% of all diabetes diagnosis in the UK and, for this patient cohort, lifestyle advice is not indicated as a treatment, beyond general health improvement measures.^22^ Finally, self-reported information in the HSE may be biased if participants do not recall information appropriately or because of social desirability bias.

### Comparison with existing literature

This study contributes new evidence to the growing body of literature suggesting that the prevalence of receiving lifestyle advice for hypertension or diabetes is low, despite lifestyle advice being recommended in clinical guidelines as the first-line intervention for both conditions.^23^^,^^24^ Research from the Netherlands has shown that lifestyle advice was provided in only 40% of hypertension-related primary care consultations,^14^ and data from Australia showed that less than a third of patients with hypertension received advice to reduce salt intake.^15^ In one study in the UK of uncomplicated stage 1 hypertension, only a third of patients received lifestyle advice, whereas half were prescribed antihypertensive medication.^11^ For those with type 2 diabetes, studies from Ghana and the US found that just over half of patients receive some form of lifestyle advice.^12^^,^^13^

In the recording of lifestyle advice provision by healthcare professionals, there was a marked increase in the proportion of patients with hypertension recorded by GPs as receiving lifestyle advice between 2009 and 2013. This coincides with both the introduction of the NHS health check programme in April 2009^25^ as well as changes to QOF indicators in April 2009 that encouraged advice for physical activity, smoking cessation, safe alcohol consumption, and healthy diet.^26^ There was a marked decline in 2014^27^ after this indicator was removed. The trend in patients’ reports of hypertension-related advice did not change during this period. The impact of this QOF indicator on the recording of lifestyle advice for patients with hypertension has previously been shown in a time series analysis from the Clinical Practice Research Datalink.^11^

Furthermore, the decline in the recorded delivery of lifestyle advice for patients with type 2 diabetes from 2013 may partly be explained by the retirement of a QOF indicator supporting the need for patients to have a dietary review by a suitably qualified professional in the preceding 12 months.^28^ Again no corresponding change is noted in patient reports from the HSE. These examples imply that changes in QOF indicators influence GP recording behaviour, but the apparent lack of corroboration from patients’ reports raises concerns that this may not reflect improved performance in delivery of meaningful or helpful support for behavioural management of these conditions.^11^

The finding that individuals with diabetes who were overweight or with obesity are less likely to receive lifestyle advice is unexpected, especially given the growing attention to the importance of weight management to treat type 2 diabetes.^9^ This result contrasts with a previous study in the US where increasing BMI was positively associated with receiving advice to exercise more among those with diabetes.^29^ However, a smaller study from Ghana of 378 patients with type 2 diabetes found no significant association between receipt of weight management counselling and weight status.^13^ It may be that these patients are referred instead to structured educational programmes such as Diabetes Education and Self-Management for Ongoing and Newly Diagnosed (DESMOND) and X-PERT where they receive lifestyle advice. Read codes for these types of diabetes education programmes were excluded from the analysis as they are general educational programmes where healthcare professionals in primary care could theoretically refer patients without directly discussing lifestyle advice.

There is relatively little research investigating the likelihood of receiving lifestyle advice for chronic diseases in relation to demographic characteristics and, as observed in the current study when comparing trends for hypertension and diabetes, the evidence is inconsistent. A previous US study using data from the National Health and Nutrition Examination Survey (NHANES) found that males and black patients were more likely to receive lifestyle counselling for hypertension than females and white patients.^30^ Another study using NHANES data found that males and white patients who were overweight or with obesity but no chronic health conditions were less likely to receive lifestyle counselling than other ethnicities.^31^ It is important to ensure that inconsistencies in the offer of lifestyle advice do not contribute to health inequalities.

### Implications for practice

Despite clinical guidelines recommending lifestyle advice and clear evidence showing the benefit of lifestyle modification to treat diabetes or hypertension, only a minority of patients with hypertension or diabetes in England are receiving lifestyle advice whereas a much larger proportion of them are receiving medication for hypertension or diabetes.

Although increasing somewhat, most patients are still missing out on opportunities to improve the management of their condition and reduce the risk of cardiovascular disease. Guidelines and evidence by themselves, and probably the QOF, have proved insufficient to change clinical behaviour and further support for effective implementation is needed to realise the potential benefits.

## Data

To guarantee the confidentiality of personal and health information, only the authors have had access to the data during the study in accordance with the relevant licence agreements. Access to the QResearch data is according to the information on the QResearch website (https://www.qresearch.org/). Access to Health Survey for England data is publicly available following registration with the UK Data Service. More information can be found on the NHS Digital website (https://digital.nhs.uk/data-and-information/publications/statistical/health-survey-for-england).
